# Impacts of Heat Stress-Induced Oxidative Stress on the Milk Protein Biosynthesis of Dairy Cows

**DOI:** 10.3390/ani11030726

**Published:** 2021-03-07

**Authors:** Zitai Guo, Shengtao Gao, Jialiang Ouyang, Lu Ma, Dengpan Bu

**Affiliations:** 1 State Key Laboratory of Animal Nutrition, Institute of Animal Sciences, Chinese Academy of Agricultural Sciences, Beijing 100193, China; i.am@guozitai.com (Z.G.); gaoshengtao1990@163.com (S.G.); 2 College of Animal Science and Technology, Yangzhou University, Yangzhou 225009, China; jialiangouyangyz@126.com; 3 Joint Laboratory on Integrated Crop-Tree-Livestock Systems of the Chinese Academy of Agricultural Sciences (CAAS), Ethiopian Institute of Agricultural Research (EIAR) and World Agroforestry Center (ICRAF), Beijing 100193, China

**Keywords:** heat stress, antioxidant, milk protein synthesis, apoptosis, ROS, cow, mitochondria

## Abstract

**Simple Summary:**

Heat stress (HS) of dairy cows affects milk protein synthesis partially due to a decline in appetite and dry matter intake (DMI). Several published studies indicate that HS causes oxidative stress (OS) with high levels of free radicals in tissues. Furthermore, the involvement of reactive oxygen species (ROS) in inducing apoptosis, in reducing mammary epithelial cell numbers, and in endocrine disruptions are proposed as the main mechanisms by which HS-induced OS modifies milk protein synthesis. However, challenges remain in determining the levels of apoptosis in vivo, as well as the tracking of the sources of ROS formation. Therefore, further investigations are required.

**Abstract:**

Heat stress (HS) is one of the most important factors posing harm to the economic wellbeing of dairy industries, as it reduces milk yield as well as milk protein content. Recent studies suggest that HS participates in the induction of tissue oxidative stress (OS), as elevated levels of reactive oxygen species (ROS) and mitochondrial dysfunction were observed in dairy cows exposed to hot conditions. The OS induced by HS likely contributes to the reduction in milk protein content, since insulin resistance and apoptosis are promoted by OS and are negatively associated with the synthesis of milk proteins. The apoptosis in the mammary gland directly decreases the amount of mammary epithelial cells, while the insulin resistance affects the regulation of insulin on mTOR pathways. To alleviate OS damages, strategies including antioxidants supplementation have been adopted, but caution needs to be applied as an inappropriate supplement with antioxidants can be harmful. Furthermore, the complete mechanisms by which HS induces OS and OS influences milk protein synthesis are still unclear and further investigation is needed.

## 1. Introduction

Exposure of farm animals to high summer environmental temperatures is globally accepted to negatively affect animal husbandry, and no livestock is so more affected than dairy cows. Generally, dairy cows suffer from heat stress (HS) once the heat accumulated surpasses the animals’ capacity to dissipate this heat [1]. HS can be defined as the symptom of nonspecific immune response exhibited by animals in hot conditions according to the stress theory proposed by Selye [2], and it negatively impacts the productive traits of livestock. For dairy cows, HS could damage their productive traits including milk quality and milk yield [3].In China, an analysis of eight major milk producing provinces indicated that hot conditions in summer were associated with substantially lower milk production of dairy cows than at other times of the year [4], which causes economic losses in the dairy industry. Furthermore, the production of milk protein, an important quality parameter, is reduced by HS. [5,6,7] In many studies, this reduction was mainly attributed to a decline in feed intake [8]. However, with the assistance of pair-feeding studies, recent HS research indicated that decreased feed intake can only partially account for the reduction in both milk production and milk protein yield [7,9,10], suggesting additional mechanisms may exist in HS to directly disturb milk protein synthesis.

Reactive oxygen species (ROS) include superoxide anions(O_2_^−^), hydrogen peroxide (H_2_O_2_), free hydroxyl radicals (OH) [11], and other products of aerobic metabolism [12]. These metabolites have dual functions in body regulation. On the one hand, ROS are related to maintaining the immune systems, for instance ROS can assist immunocytes in removing pathogens [13], and some others, such as H_2_O_2_, can act as second messengers for intercellular information exchange through redox signals [14]. On the other hand, increased ROS aggravates metabolic dysfunctions and even cause cell death, as ROS can modify highly reactive cellular macromolecules including organism lipids, proteins, and deoxyribonucleic acid (DNA) [15,16]. To prevent oxidative damage, animals are equipped with a defense mechanism, namely antioxidants, to counterbalance the effects of ROS, and these important compounds can be divided into enzymatic as well as nonenzymatic antioxidants [17]. When ROS are produced faster than the neutralizing ability of antioxidants, metabolic dysfunctions finally cause oxidative stress (OS) [12,18].

The involvement of HS as an inducer of OS in dairy cows has been acknowledged [19,20,21]. The decreased content of antioxidants in Holstein cows exposed to hot conditions suggests that HS participates in enhancing the formation of ROS [22,23,24]. This is notable, considering that DMI being reduced by HS may help to counteract OS by decreasing the absorption of some compounds from the digestive system. Since it is reported that OS participates in mTOR pathway regulation, as well as mediating apoptosis in mammary epithelial cells [25,26,27], the imbalance between ROS and antioxidants caused by HS may contribute to the reduction in milk protein through these two mechanisms. However, only a few studies have focused on the role of OS under HS on milk synthesis to comprehensively explain the effect of HS on milk protein synthesis of dairy cows. Hence, the current review aims to illustrate how OS induced by HS takes part in this process.

## 2. Heat Stress Associates with the Oxidative Stress in Body

### 2.1. The Determination of Oxidative Status in HS Cows

ROS are metabolites produced in the process of mitochondrial electron transport chain reaction [28], and these reactions are mostly mediated through redox chemistry of metal ions. Generally, transition metals in the body except for copper (Cu) contain an electron in the outermost shell while copper can easily loss or gain electrons even though it has a full outer shell [29]. Thus, iron as well as copper in the body become the common catalysts to induce oxidation reaction, as the electron can be considered as a free radical. The reduced forms of metal ions participate in a Fenton reaction where HO˙ is produced, then the ions in oxidative forms can return back to reduced forms by the Haber-Weiss reaction and participate in the Fenton reaction again [30,31]. As a result, redox active metals are likely to lead to the formation of OH˙.

Fenton reaction:

Metal^(n+1)^ + H_2_O_2_ → Metal^(n+1)+^ + HO˙ + H_2_O (1)

Haber-Weiss reaction

Metal^(n+1)+^ + 2O^2−^˙/AA/GSH → Metal^(n+1)^ + O_2_/AA˙/GSSG (2)

AA = ascorbic acid
GSH = reduced glutathione
GSSG = Oxidized glutathione

Obtaining direct measurements of ROS in vivo are difficult since the ROS has a very short half-life [32]. A few techniques including electron spin resonance (ESR) can detect free radicals directly relying on unpaired electrons of free radicals [33]. Unpaired electrons of free radicals behave as magnet so they can align either in a parallel or antiparallel manner when exposed to external magnetic fields [34]. Therefore, the two energy levels free radicals created can be varied and absorbed. Hence, free radicals can be identified by the absorption spectrum obtained via ESR [29]. However, the steady-state requirement limits the use of ESR in vivo; even though radicals can be accumulated to the level that does permit detection by ESR and spin trapping, the metabolism of the trap as well as the delicate nature of the trap itself may produce radicals in its absence [35]. Thus, a combination of ESR and spin trapping should be carefully used in vivo.

Indirect methods are widely selected for these tasks because the measuring effects caused by ROS instead of the total amount of generated ROS are easier to use. The techniques including fingerprinting methods and the measurements of antioxidant defense systems have been developed to detect oxidative status [29]. Fingerprinting is an alternative to trapping; its aim is to measure and quantify products of damage caused by ROS such as proteins, lipids, and DNA, and these end-products should specifically consider oxidative damage [36,37]. The determination of an antioxidant defense system is mainly achieved by measuring the enzymes such as superoxide dismutase (SOD) and catalase; there are two approaches available for this assay: one is to measure all the individual antioxidants recognized, while the other approach is to measure the capacity or activity of antioxidants by subjecting them to a controlled OS condition [38]. However, the synergistic effects among antioxidants and the influence of unknown antioxidant substances causes measuring the individual antioxidants to be time consuming and technically demanding to implement. In fact, the measurement of all kind of ROS within cells or overall antioxidative status at one specific time can be quite difficult since none of the parameters can predict the development of diseases induced by prolonged OS [39]. Therefore, it is important to employ multiple detection assays and consider the measurement of different parameters to enhance a method’s validity.

### 2.2. Heat Stress Contributes to the Overproduction of ROS

Oxidative stress occurs when the balance between pro-oxidants and antioxidants is disturbed and leads to an elevation in concentrations of free radicals and ROS [40,41]. ROS are metabolites produced in the process of mitochondrial electron transport chain reaction [28] and the vast majority are related to an increase in cellular aerobic respiration [12,28]. Various oxidase pathways such as nicotinamide adenine dinucleotide phosphate oxidase (NADPH oxidase) can generate ROS [42,43] when NADPH is converted to NADP^+^. The ROS produced in mitochondria are mainly superoxide anions, which can go on to generate hydrogen peroxides under the catalysis of superoxide dismutase (SOD) in the inner membrane of the mitochondria [44].

Several studies have demonstrated that HS promotes OS and ROS production [23,45], while HS can activate NADPH oxidase and increase the ratio of NADP^+^/NADPH, and it has been shown that heat treatment can enhance this ratio without any changes to NADPH subunits or the NADPH oxidase 1 (NOX1) mRNA expression [46]. Furthermore, the knockdown of NOX1 significantly inhibited ROS production, suggesting that NOX1 plays an important role in the process of HS inducing ROS [46].

HS leads to an overproduction of transition metal ions (TMI) by increasing the rate of iron release from ferritin [47]. TMI can donate electron to oxygen, forming superoxide anion and hydrogen peroxide [48,49], while the electron capture by oxygen is also conductive to the formation of H_2_O_2_, and hydroxyl radicals via the Fenton reaction, as mentioned above [50].

Hydrogen peroxides are able to diffuse across cell membranes because of their small size and relatively benign reactivity, and they can go on to modify different proteins including phosphatases, transcription factors, ion channels, etc. [51]. While hydroxyl radicals have a very-short half-life, they can react with many molecules that are extremely reactive [52]. Hence, an uncontrolled elevated level of ROS concentrations may induce free radical-mediated chain reactions which indiscriminately target and damage proteins, lipids, polysaccharides, and DNA [53,54,55,56].

### 2.3. Heat Stress Causes the Oxidative Stress by Inducing Mitochondrial Dysfunction

Previous studies have shown that Holstein cows under heat exposure have a higher level of thiobarbituric acid-reactive species and malondialdehyde than cows not exposed to heat stress [57,58]. Since these two factors are the major products of polyunsaturated fatty acid (PUFA) peroxidation [59], HS may be strongly associated with lipoperoxidation of mitochondrial membranes, which is another important source of free radicals production [41]. Furthermore, studies in nonneuronal cells and isolated mitochondria demonstrated that the amounts of energy produced by mitochondria are disrupted by HS [60]. Hyperthermia increases the permeability of the mitochondrial inner membrane and impairs oxidative phosphorylation [61,62]. The damage reported in mitochondria isolated from the hearts of rats treated with hyperthermia included a reduction in ATP synthesis, and a greater tendency to open mitochondrial permeability transition pores [61].

The heat stress response of cells is a highly conserved cascade of protein activation and gene expression changes which are regulated by heat shock transcription factors and that participate in the defense of stress damage [63]. Heat shock transcription factors are commonly found in organisms and have extensive homology, and are separated and activated from heat shock proteins (HSPs) after being stimulated by ROS, viruses, hyperthermia, and other factors [64]. The complexes formed can bind to the promoter region of heat stress elements, thereby regulating the transcription of HSPs [65,66]. Heat shock proteins are a protein super family, for which the function of many members involves molecular chaperones that function to stabilize other proteins that have been damaged by cell stress. These molecules are in the weight range from less than 10 KD to more than 110 KD [67] and both of their cellular locations and specific functions are different [68]. For example, the HSP70 family includes HSC73, HSP72, GRP78, and GRP75 that are able to assist with degrading unstable proteins as well as regulating the activity of transcription factors [69].

As summarized above, mitochondria are thermosensitive [70] and as such the effects of HS are on their most basic function, namely respiration [71]. Mitochondrial respiration is defined as a continuous reaction system composed of a series of hydrogen transfer and electron transfer reactions that are arranged in a specific order [72]. Energy in the form of electron flow source from the respiratory chain is converted into a H^+^ gradient through the inner mitochondrial membrane [73]. Then this H^+^ gradient dissipates through the adenosine triphosphate (ATP) synthase complex producing ATP [74]. The amount of ATP in cells is regulated by both mitochondrial and glycolytic ATP synthesis. While HS can inhibit mitochondrial ATP synthesis and result in the dysfunction of the electron transport chain [75], it has also been shown that HS can inactivate the complex I of the respiratory chain without affecting other complexes [76]. Consequently, the rate of electron flow through the electron transport chain is slowed down under HS, resulting in a decreased oxygen uptake but a increased formation concentration of O_2_^−^ [77], and all of these factors above are correlated with the OS.

## 3. Oxidative Stress Induced by Heat Stress Reduces Milk Protein Synthesis

### 3.1. Heat Stress Directly Affects the Synthesis of Milk Protein

Animals suffer an HS decrease in metabolic heat production to maintain homeothermy, often causing decreased productivity. For dairy cows, HS decreases milk yield as well as milk protein, and this is partly due to the shortage of synthesis precursors in mammary glands [7,24]. Amino acids and glucoses are precursors for the synthesis of casein (the main component of milk protein), while rumen microbial protein is the main source of amino acids entering mammary glands. Hence the reduction in milk protein is highly correlated with decreased DMI under HS. However, recent studies have shown that the role of DMI cannot totally explain the negative effects of HS on milk protein synthesis [9]. The liver and mammary glands act cooperatively for the provision and utilization of milk protein precursors, while research using isotope labeling showed that HS may improve the ability of tissues (apart from mammary glands) to utilize glucose instead of the ability of hepatic gluconeogenesis [78]. Studies showed that HS can affect the lipid and carbohydrates metabolisms in cattle [79]. Gao et al. [7] conducted a pair-feed trial and found that HS significantly reduced the total amino acid and the content of amino acids including threonine, serine, glycine, cysteine, isoleucine, lysine, and arginine in blood compared to thermal neutral levels. Eventually, nutrient redistribution induced by HS causes the reduction in supplements of precursors of casein in the mammary gland and results in decreased milk protein synthesis.

Furthermore, signaling pathways dominated by Janus kinase 2/Signal transducers and activators of transcription (JAK2/STAT5) as well as mechanistic target of rapamycin (mTOR) are involved in the regulation of milk protein synthesis [80]. Related studies have found that HS inhibited the metabolic activity in mammary gland through RNA seq combined with data independent acquisition (DIA), while the inhibited genes relative to amino acid and glucose transporter reduces the overall casein synthesis ability [81]. Therefore, HS directly inhibits the synthesis of milk protein and results in the reduction of its content in milk.

### 3.2. Heat Stress Mediates Mammary Epithelial Cells Apoptosis through Oxidative Stress

Apoptosis is a process of energy dependent programmed cells death characterized by the specific degradation of cellular DNA. This process is regulated by specific genes and is morphologically manifested as nuclear pyknosis, cell membrane foaming, and apoptotic body formation and ends up with cells break down without any obvious lysis phenomenon [82]. Apoptosis can be activated by either intrinsic or extrinsic pathways, both of which are mediated by the caspase family of cysteine proteases [83]. The extrinsic pathway is induced by the activation of death receptors on the cell membrane, including factor associated suicide (FAS) and tumor necrosis factor receptor 1 (TNFR1) [84]. Meanwhile, the intrinsic pathway involves the mitochondrial and a complex of factors including stress conditions, chemical damages, and pharmaceutical damages [85].

Heat Stress can directly activate the mitochondrial pathway to mediate apoptosis [86]. Hyperthermia significantly decreases cell viability as well as resulting in abnormal mitochondrial morphology, which is an early signal of apoptosis [87]. The pro-apoptotic molecules are transferred to mitochondria and trigger apoptosis through the B cell lymphocytic-leukemia proto-oncogene (Bcl-2), while the Bcl-2 family includes both anti-apoptotic members like Bcl-2 and Bcl-xL and pro-apoptotic members such as Bax and Bak [88]. Under HS conditions, there are two mechanisms affecting outer mitochondrial membrane permeabilization to cytochrome c [89]. The first one relies on the outer mitochondrial membrane pores being regulated by Bcl-2 family members, for example, Bax/Bak oligomeric pores and Bax (Bak) voltage dependent anion channel hybrids [90,91,92]. The second mechanism involves mitochondrial Ca2+ overload caused by hyperthermia, where the overload results in persistent opening of the mitochondrial permeability transition pores, resulting in the nonspecific rupture of the outer mitochondrial membranes [93,94]. The rupture causes the releasing of cytochrome c, which binds to apoptotic protease activating factor 1 (Apaf-1) and forms the apoptosome. Hence, the apoptosome activates the initiator caspase-9 and its downstream effectors caspases-3 and caspase-7 [95], which then induces the subsequent apoptosis process. Furthermore, the overload of ROS or mitochondrial Ca^2+^ seems to assist the release of apoptotic cytochrome c, as was reported in some previous studies [96,97].

Since cellular stress, including OS, induces mitochondria to generate ROS [98], this has serious consequences including causing oxidative damages for mitochondrial DNA (mtDNA) [99]. Elevated levels of superoxide anions and hydroxyl radicals are considered to not only be associated with mtDNA damages, but also serve a major role in cell apoptosis [100]. However, the mechanism of mtDNA damage mediating apoptotic signaling is still not properly understood [100]. Furthermore, the involvement of ROS in apoptosis is controversial. The generation of free radicals or depletion of antioxidants induce apoptosis [101], but the apoptosis process can also occur at low oxygen tension (as Bcl-2 can show in the absence of ROS) [102]. The glutathione redox pair is an index of OS, hence a reduction in glutathione (GSH)/oxidized glutathione (GSSG) is proposed as a regulator of enzyme activities [103]. But the glutathione redox pair cannot evaluate the participance of OS in apoptosis process, as the glutathione depletion in apoptosis is due to an increased efflux of the reduced form; hence, this type of loss is nonoxidative [104]. As mentioned before, the regulation of cytochrome c released by mitochondria is important in apoptosis induction. That mitochondrial permeability transition channels (MPT) are independent of free radicals can explain the results obtained in both anaerobiosis and in aerobiosis. Related reports indicated that OS can regulate cytochrome c to be released from mitochondria via the mitochondrial permeability transition channels [25], suggesting that OS is a cause instead of a consequence in the apoptotic process, at least under aerobic conditions.

The reduction in the amount of lactating mammary epithelial cells can affect the synthesis of milk protein. Results of studies in vitro have shown that hyperthermia as well as the elevated OS contribute to the apoptosis of lactating mammary epithelial cells [62,105]. However, due to the limitation of cell apoptosis detection technology, it is still not yet fully proven that HS and its induced OS can induce apoptosis of mammary epithelial cells in vivo.

### 3.3. Oxidative Stress Results in Insulin Resistance and Associates with mTOR Pathway Regulation of Milk Protein Synthesis

The synthesis of milk protein is regulated by hormones including prolactin (PRL), glucocorticoids, thyroxine, and insulin [106], while the process of all protein synthesis, especially translation, is mediated by the mammalian target of the rapamycin (mTOR) pathway [107]. The mTOR pathway in mammals is a highly conserved and plays an important role in responding to external environmental signals of metabolism, growth, proliferation, and survival. mTOR is involved in the formation of mTORc1 and mTORc2 and serves as the catalytic core of these two signal complexes [108]. mTORc1 can not only regulate the synthesis of ribosome proteins, but exhibits a higher sensitivity to rapamycin than mTORc2, which plays an important role in regulating cell growth and reproduction [109,110]. The substrates that participate in mTORc1 regulation include ribosomal protein S6 kinases (S6K1 and S6K2) and eukaryotic initiation factor-4E-binding proteins (4EBPs) [111,112]. Also, 4EBP can inhibit the activity of eukaryotic initiation factor 4E (eIF4E), while the latter one can bind to the cap structure of mRNA. Furthermore, the site of 4EBP_S_ binds to eIF4E is also the binding site of eIF4E and eIF4G, which affects the formation of translation initiation complexes [112]. mTORc1 can phosphorylate sites of 4EBPs to dissociate them from eIF4E, thereby promoting translation [112]. S6K1,a significant member of the AGC protein kinase family, can phosphorylate eEF2 kinase which in turn influences the initiation and extension of mRNA translation [113]. eEF2 is the translation elongation factor in mammalian cells, which assists with promoting the transfer of peptidyl RNA from A to P during translation and the subsequent formation of peptides [114]. eEF2 kinase specifically catalyzes the phosphorylation of eEF2 to inactivate the eEF2. Moreover, mTOR phosphorylates eEF2 kinase to inactivate the phosphorylated eEF2 and induces the dephosphorylation of eEF2, so as to promote translation elongation [115].

Insulin participates in the regulation of the mTOR signal pathway, as it can activate upstream protein kinase B (PKB) and further act on mTOR [26,116]. Insulin enhances phosphorylation levels of S6K1 as well as 4EBP1, promoting the transcription of mTOR signaling pathways; insulin can also activate eIF2 to further decrease the inhibitory effect of the massive activation of PKB [117]. Consequently, insulin is beneficial to protein synthesis and translation. However, former studies showed that insulin alone has an insignificant effect on the mTOR pathway, as it accelerates the transcription of milk protein genes and protein translation when interacting with other nutrients [118]. For example, growth hormone (GH) and insulin can synergistically promote protein synthesis and this effect is more intensive than that of insulin or GH alone [119]. Furthermore, as 80 percent of the glucose in body can be utilized by mammary glands, a certain concentration of insulin will reduce the uptake of metabolic substrates in other tissues [120]. This kind of nutrient distribution provides more synthesis precursors to the mammary gland, hence promoting the synthesis of milk protein. Additionally, one mouse research study showed that insulin can directly stimulate the expression of 28 protein synthesis genes, including 4 types of casein [121].

While OS has negative effects on milk production as described above, OS has also been shown to induce the insulin resistance (IR), impaired insulin secretion, and diabetes [27]. IR is defined as a state whereby normal concentrations of insulin are unable to stimulate adequate biological responses in insulin-sensitive tissues [122]. The accumulation of reactive molecules leads to the activation of multiple serine kinase cascades and/or inhibition of PTPases [123,124]. The insulin signaling pathway provides potential targets of these activated kinases, including the insulin receptor and the family of insulin receptor substrate (IRS) [125]. The ascended serine phosphorylation of IRS-1 and -2 could reduce those extent of tyrosine phosphorylation, which is consistent with the attenuation of insulin activity [126,127]. A published study found that H_2_O_2_ caused an increase in serine phosphorylation of IRS-1 as well as -2 but also decreased IRS-1 in 3T3-L1 adipocytes [128]. The motivated serine/ threonine phosphorylation accelerates the degradation of IRS-1 and then results in IR. Former research of dairy cows found that the tissues of liver and mammary glands contained a cooperative mechanism for the distribution and utilization of precursors required by milk protein synthesis [78]. Insulin has a multitude of effects on various metabolic pathways in different insulin-sensitive tissues. Except for the promoting protein synthesis as mentioned above, insulin participates in the suppression of glycogenolysis while stimulating glycolysis in liver and skeletal muscle [129,130]. The decreased biological response of insulin caused by IR affects the stimulation of protein synthesis induced by insulin via the mTOR pathway. Furthermore, effects of insulin on glycolysis as well as glycogenolysis are inhibited. These inhibitions together with HS damages contribute to the negative energy balance, triggering the mobilization of amino acids in blood for deamination reaction to meet energy requirement of organs. Thus, this redistribution reduces the supply of precursors and end up decreasing milk protein synthesis.

## 4. Strategies for Alleviating Heat Stress-Induced Oxidative Stress

To alleviate the OS induced by HS, strategies to reduce OS by neutralizing the overproduction of ROS or inhibiting its formation should be considered first [131,132]. But methods to reduce HS directly seems to be more effective in reducing its induced OS by either enhancing heat losses or lowering condition temperatures. These attempts of environmental modifications can be classified as shading, air cooling, ventilation, and spraying according to the different realization approach, and are usually combined to adopt in different practices [133]. For example, physical cooling strategies of the combination of spraying and ventilation are commonly used in dairy barns, in which the roof insulation was equipped as well. However, except for using air condition to cool cows, most parts of strategies cannot alter air temperature nor relative humidity in barns, which increase the difficulty of assessing the alleviating effects [134]. Few methods have been systematically evaluated. For example, in spraying, selecting a flow rate of 1.3 L/min to spray cows was reported to have the highest efficiency, while excessive water did not further reduce HS [135,136]; in shading, the total heat load in open barns can be reduced by 30% or more with a well-designed roof [137]; in ventilation, the addition of low volume and high speed fans was proved to significantly decrease the rectal temperature as well as respiration rate of dairy cows [138]. But considering that the climate can vary greatly in different areas, cooling strategies should be properly configured for different conditions and be cautiously adopted.

Apart from alleviating heat stress of dairy cows using direct cooling methods (fans and sprays), antioxidants supplementation has been proven useful in HS situations to reduce the occurrence of udder infection as well as improve milk protein content [139]. Numerous antioxidants contribute to the defense mechanism with their own specific functions [140]. A variety of antioxidants including vitamins, carotenoids, polyphenolics, and trace elements can be added as dietary supplements [141,142]. Among feed-derived antioxidants, vitamin E and selenium (Se) are always considered as the primary factors which contribute to defending against OS [143,144]. Vitamin E is in a group of fat-soluble vitamins including four tocopherols and four tocotrienols [145], which are hypothesized to act catalytically, being efficiently reduced from their free radical forms to their native states [146]. Specifically, vitamin E can transfer hydrogen atoms (H) to free radicals, as the O-H bond in tocopherol located at 323 KJ/mol is about 10% weaker than most of phenols [147,148]. This weak bond allows vitamin E to contribute H to hydrogen superoxide and other free radicals, hence the ROS are neutralized and damage is minimized [147]. Through the oxidation-reduction reaction of hydrogen donors such as vitamin C, the tocopherol radicals that are thus generated are reused as tocopherols. Since vitamin E is fat-soluble, it can also be incorporated into cell membranes to protect cells from oxidative damage [149]. Compared with vitamin E, Se is considered to be a more important defense system. This trace element participates in the synthesis of selenoproteins and is related to the maintenance of redox balance [150]. Studies indicated that nineteen selenoproteins in cows are involved in antioxidant defenses while a total of 25 selenoproteins are identified to participate in body regulations [151]. Some of the 19 antioxidative selenoproteins are members of glutathione peroxidase (GSH-Px) and thioredoxin reductases (TrxR) [151]. GSH-Px has the function of reducing lipid peroxides to alcohols and reducing free hydrogen peroxide to water, while catalyzing the conversion of glutathione to its oxidized form [152]. TrxR is the unique enzyme catalyzing the reduction of thioredoxin [152], while the thioredoxin system induces the formation of reduced disulfide bonds in cells and can alleviate the OS originated from oxygen metabolism by taking electrons from NADPH [153]. Consequently, the supplementation of diets with antioxidants contributes to build up the defense system and accordingly alleviates the extent of OS.

The preceding paragraph detailed the inclusion of the non-enzymatic antioxidants in diets, but the supplementation of plant natural extracts including polyphenols, flavonoids, tannins, and gallic acid has been attracting more attention in recent years due to their high efficiency as well as low residuals [154,155,156]. Among those different types of extracts tannins are the most-studied compounds [157], as they have the ability to scavenge free radicals by donating electrons to make those structures more stable and less toxic [158]. Tannins are classified into two groups: hydrolysable and condensed tannins [159]. A number of condensed as well as hydrolysable tannins were evaluated and reported to have the effects on super-oxide radicals, hydroxyl radicals, and nitric oxide [160,161,162].

The inappropriate use of antioxidant supplements is possible and may lead to the antioxidative imbalance and result in carcinogenesis [163]. As mentioned, ROS has a dual effect in tissues, hence slight OS is sometimes beneficial for the organism. In this case, antioxidant supplementation is not as beneficial as expected, for it may reduce some ROS which act as signaling molecules in important pathways. Therefore, it is always essential to keep the balance between ROS and antioxidants, no matter which kinds of strategies are adopted.

## 5. Conclusions

Heat stress causes an overproduction of ROS and mitochondrial dysfunction, leading to oxidative stress in dairy cows. Current published studies strongly suggest that oxidative stress as a result of heat stress contributes to a reduction in milk protein. This is caused by an increase in apoptosis in mammary gland tissues that directly reduces the number of mammary epithelial cells, while elevated levels of free radicals also damage milk protein synthesis by regulating signaling pathways. The dietary supplementation of antioxidants has been adopted to alleviate OS including that induced by HS, and this is a strategy that has been proven to be useful in certain circumstances. However, current technology has not been able to establish the real extent of apoptosis in mammary epithelial cells in vivo nor the accurate source of free radical formation in all situations (e.g., aerobic or anaerobic conditions). Further advances are needed to fully understand the effects of HS-induced OS on milk protein reduction, which have the potential to facilitate new methods to reduce the effects of high environmental temperatures on poor milk production in dairy cows.

## Data Availability

All data used in this study are included in the main text.
